# Transcriptome Analysis of the Japanese Pine Sawyer Beetle, *Monochamus alternatus*, Infected with the Entomopathogenic Fungus *Metarhizium anisopliae* JEF-197

**DOI:** 10.3390/jof7050373

**Published:** 2021-05-10

**Authors:** Jong-Cheol Kim, Mi-Rong Lee, Sihyeon Kim, So-Eun Park, Se-Jin Lee, Tae-Young Shin, Woo-Jin Kim, Jaesu Kim

**Affiliations:** 1 Department of Agricultural Biology, College of Agriculture & Life Sciences, Jeonbuk National University, Jeonju 54896, Korea; jc.kim124@gmail.com (J.-C.K.); mimilee248@naver.com (M.-R.L.); kdkim22@naver.com (S.K.); pse0330@naver.com (S.-E.P.); tyshin@jbnu.ac.kr (T.-Y.S.); 2 Department of Agricultural Life Science, Sunchon National University, Suncheon 57922, Korea; sejinlee@scnu.ac.kr; 3 Department of Agricultural Convergence Technology, Jeonbuk National University, Jeonju 54596, Korea

**Keywords:** bioinformatics, *Metarhizium anisopliae*, mode of action, *Monochamus alternatus*, RNA sequencing

## Abstract

The Japanese pine sawyer (JPS) beetle, *Monochamus alternatus* Hope (Coleoptera: Cerambycidae), damages pine trees and transmits the pine wilt nematode, *Bursaphelenchus xylophilus* Nickle. Chemical agents have been used to control JPS beetle, but due to various issues, efforts are being made to replace these chemical agents with entomopathogenic fungi. We investigated the expression of immune-related genes in JPS beetle in response to infection with JEF-197, a *Metarhizium anisopliae* isolate, using RNA-seq. RNA samples were obtained from JEF-197, JPS adults treated with JEF-197, and non-treated JPS adults on the 8th day after fungal treatment, and RNA-seq was performed using Illumina sequencing. JPS beetle transcriptome was assembled *de novo* and differentially expressed gene (DEG) analysis was performed. There were 719 and 1953 up- and downregulated unigenes upon JEF-197 infection, respectively. Upregulated contigs included genes involved in RNA transport, ribosome biogenesis in eukaryotes, spliceosome-related genes, and genes involved in immune-related signaling pathways such as the Toll and Imd pathways. Forty-two fungal DEGs related to energy and protein metabolism were upregulated, and genes involved in the stress response were also upregulated in the infected JPS beetles. Together, our results indicate that infection of JPS beetles by JEF-197 induces the expression of immune-related genes.

## 1. Introduction

The Japanese pine sawyer (JPS) beetle, *Monochamus alternatus* Hope (Coleoptera: Cerambycidae), is a major pest that causes serious damage to pine trees by mediating transmission of the pine wilt nematode, *Bursaphelenchus xylophilus* Nickle (Aphelenchida: Aphelenchoididae) that causes pine wilt disease [1]. This beetle has spread to East Asian countries including Korea, Japan, and China, in addition to Portugal in Europe [2,3]. Disease-causing nematodes or insects that mediate the transmission of these nematodes need to be controlled to prevent the spread of pine wilt disease. The main means of controlling JPS beetles are chemical agents such as neonicotinoids, fenitrothions, thiacloprids, and thiamethoxams [4,5]. However, these chemical agents are toxic to the environment and can induce insect resistance. Therefore, natural microbial materials have been investigated as alternatives to these chemical agents [6,7]. Among natural microbial agents, entomopathogenic fungi have attracted great interest as environmentally sound biopesticides [8]. The entomopathogenic fungus *Beauveria bassiana* was developed as a biopesticide in Japan to control JPS beetles [9,10,11,12], and in Portugal, it has been used to control *M. galloprovincialis*, another allied insect species that transmits the pine wilt nematode [13]. Several other countries are also conducting research into entomopathogenic fungi as control agents for nematode vectors. Previously, we confirmed that this fungus is highly virulent against JPS beetles [14,15].

Fungal-based biopesticides, however, are slower at controlling insects than chemical pesticides. Therefore, research into the responses of insects to fungal toxins is required to develop methods to accelerate insect host killing [16,17]. The immune reaction of insect hosts begins with immune system elements such as the complement system, immune cells, and antimicrobial proteins that recognize pathogen-associated molecular patterns (PAMPs). Insects, however, can become resistant to pathogen infection due to evolved immune system responses. Cellular reactions are mediated by hemocytes that initiate phagocytosis, encapsulation, and/or modulation of pathogens. Moreover, the humoral response promotes the production of antimicrobial peptides, coagulation, and melanization [18,19,20,21,22]. The iridoid monoterpene, a major component of antifungal secretion, is used by the mustard leaf beetle, *Phaedon cochleariae*, to inhibit the growth of *Beauveria bassiana* in vitro [23].

The insect epicuticle comprises a wax layer containing fatty acids, lipids, and sterols, and is a structurally and chemically complex tissue. Infection can begin only when the conidia of the fungus attach firmly to the outermost insect integument by hydrophobic interactions or mucilage [24,25,26]. To avoid the host immune system, some pathogens express cell surface proteins that mimic the host surface or dissociation proteases that degrade host immune proteins [27]. Fungi that penetrate the cuticle of insects induce antimicrobial activity in the cuticle matrix and fat body and defense mechanisms such as localized melanization around penetrating fungal hyphae [26,28,29]. Fungi secrete extracellular hydrolytic enzymes such as catalase, chitinase, lipase, phospholipase C, and protease to invade the insect host [30,31].

RNA-seq is an ideal method for analyzing the expression levels of a large number of genes in insects infected with fungi, and when reference genomes are not available, as is the case for JPS beetles, the transcript sequences obtained can be used for *de novo* transcriptome assembly [32]. RNA-seq not only provides information about RNA sequences and abundance, but it can also provide insights into RNA processing and regulation [33,34]. A previous study generated a gene database of JPS beetles using Illumina sequencing technology [35]. Furthermore, a unique recognition gene—namely, galectin-3—was found to be expressed at high levels in JPS adults in response to invading microorganisms but was not identified in other insects [36]. In addition, chemosensory genes, insecticide resistance-related genes, insecticide receptor genes, RNA interference genes, *Bacillus thuringiensis* (Bt) receptors, intestinal digestive enzymes, and immune-related genes have been identified in JPS beetles through transcriptome analysis [37,38]. In particular, RNA-seq analysis of the pupae of JPS beetles treated with symbiotic microorganisms and fungi was used to investigate the immune response of JPS beetles to infection. The Toll pathway was found to be involved in the immune response to the entomopathogenic fungi *B. bassiana*, and Toll and IMD signaling was involved in the immune response to the symbiotic fungi *Sporothrix* sp. These studies confirmed that insects can show immune responses to fungi [39].

In this study, we analyzed changes in gene expression in JPS beetles after treatment of *Metarhizium anisopliae* JEF-197, a fungus that was shown to have high virulence in JPS beetles in a previous study [14]. To demonstrate the potential of JEF-197 as a biopesticide, we performed semi-field experiments to target the reproducing JPS in late summer [14] and newly emerging JPS from infested trees in early summer [15], both of which target JPS adults to study the reaction of JPS adults against the entomopathogenic fungus. We studied changes in gene expression in JPS beetles during the process of JEF-197-induced death and confirmed that the expression of specific genes in JPS beetles changed significantly at specific time points after fungal treatment. Total RNA of JPS beetles was extracted at various time points, and Illumina short reads of RNA-seq libraries were obtained. A protein coding in silico cDNA library of JPS adults was constructed by *de novo* assembly, and DEGs were analyzed by mapping short reads to the in silico cDNA library. Changes in metabolism were confirmed by analyzing pathways and performing gene ontology (GO) analysis. In addition, changes in the expression levels of JPS adult immune genes upon fungal infection were assessed. Secondly, we confirmed that the expression level of specific fungal genes changed significantly during fungal infection. Candidate genes of *M. anisopliae* that potentially played a role in killing JPS adults were identified through bioinformatic analyses.

## 2. Materials and Methods

### 2.1. Japanese Pine Sawyer Beetles and the Entomopathogenic Fungus JEF-197

Adult Japanese pine sawyer (JPS) beetles, *M. alternatus* Hope, were obtained from the insect rearing company Osangkinsect Co. (http://www.osang.com, accessed on 16 April 2021, Korea). Final stage larvae were stored at 10 °C to induce dormancy. Larvae were maintained at 20 °C for 7 days for dormancy awakening and then moved to 25 °C for pupation and emergence. Rearing conditions were 60% relative humidity (RH) and a photoperiod (light:dark) of 16:8. All experiments involved JPS adults within 10 days of emergence.

The entomopathogenic fungus *Metarhizium anisopliae* isolate JEF-197 (Insect Microbiology and Biotechnology Laboratory, Jeonbuk National University, Korea), which was originally isolated from soil collected on a mountain (35°43′42” N 127°06′03” E, Wanju, Korea), was used in this study [14]. In a previous study, this fungal isolate showed high virulence against JPS adults. JEF-197 was cultured on quarter-strength Sabouraud dextrose agar (1/4 SDA, BD Difco ™, NJ, USA) at 27 °C for 14 days in the dark. Conidia were harvested from cultures grown on 1/4 SDA and resuspended in 0.03% siloxane solution (Silwet, FarmHanong, Nonsan, Korea). Concentration of the conidia suspension was assessed using a hemocytometer, and conidia were diluted to 1.0 × 10^7^ conidia/mL in 0.03% siloxane solution to prepare conidial suspensions.

### 2.2. Treatment of JPS Adults with M. anisopliae JEF-197

One JPS adult was placed in a plastic cup (lid 100 mm diameter × bottom 60 mm diameter × height 150 mm), and then sprayed either with 1 mL of the conidial suspension (1.0 × 10^7^ conidia/mL using 0.03% siloxane solution) or 1 mL 0.03% siloxane solution (non-treated control). Plastic cups with JPS adults were maintained at 25 ± 2 °C at 95% RH, and an approximately 10 cm long pine stem was placed in the dish as a food source. Mortalities of JPS adults were assessed on a daily basis. Fifteen JPS adults were included in each treatment group, and experiments were repeated in triplicate. Differences in mortality of JPS adults among groups were analyzed using *t*-tests in SPSS ver. 19.0 (SPSS Inc., Chicago, IL, USA) using a significance level of 0.05 (α), and LT_50_ was calculated by probit analysis.

### 2.3. RNA Extraction and Construction of RNA-Seq Libraries

JEF-197 fungal samples were cultured in 1/4 SDA medium and harvested on day 8 after inoculation of the medium. JPS samples were treated with the fungus as described above, and then three live JPS adults from fungal-treated and non-treated control groups were obtained on the 8th day after treatment, respectively. To analyze changes in the expression of specific immune-related genes over time, additional JPS samples were collected from fungus-treated and non-treated control groups at 6, 24, 48, 72, 96, 120, and 192 h after treatment as described above.

RNA was extracted from all samples on the 8th day after fungal treatment. Individual adult JPS beetles were placed in 15 mL conical tubes (SPL life sciences, Pocheon, Korea) with 5 mL Trizol ^TM^ reagent (Molecular Research Center Inc., Cincinnati, OH, USA). Adult JPS beetles were then ground using an iron pestle, and 400 μL of ground insect sample in Trizol reagent was transferred to a 1.5 mL microtube and ground again using an Ultra grinder BTM (Taeshin Bio Science, Namyangju, Korea). Total RNA from fungus-treated and non-treated control JPS adults was extracted using Trizol reagent according to the manufacturer’s instructions. The integrity of the extracted RNA was examined using an Agilent 2100 Bioanalyzer (Agilent Technologies, Santa Clara, CA, USA). Sequencing libraries of the samples were made using the Truseq RNA kit (Illumina, San Diego, CA, USA) according to the manufacturer’s protocol. cDNA library construction and Illumina sequencing of the samples were performed at Macrogen Corporation (Seoul, Korea). First, poly-A containing mRNA molecules were purified using poly-T oligo-attached magnetic beads followed by fragmentation into small pieces under elevated temperature with divalent cations. Cleaved RNA fragments were reverse-transcribed into first strand cDNA with random primers. This was followed by second strand cDNA synthesis using DNA polymerase I and RNase H. These cDNA fragments were subjected to an end repair process, addition of a single dATP, and ligation of sequencing adapters. Products were then purified and enriched by PCR to create the final cDNA library. All samples were sequenced on an Illumina HiSeq2000 sequencer (Illumina, San Diego, CA, USA) to generate high-throughput transcriptome sequence data with an average read length of 101 bp.

### 2.4. De Novo Transcriptome Assembly and Differentially Expressed Gene (DEG) Analysis

Three biological replicates from each group were sequenced, and the qualities of the Illumina short reads were checked using fastQC v.0.11.8 [40]. Short reads were filtered to remove low-quality sequences using the trimmomatic program [41], and JEF-197 and JPS beetle contigs were assembled using the Trinity *de novo* assembler v.2.8.5 [42] with default options. Assembled contigs with protein coding capacity were identified by TransDecoder v5.5.0 (https://github.com/TransDecoder, accessed on 16 April 2021). The numbers of short reads that mapped to contigs were quantified by Kallisto v.0.45.0 [43] to calculate transcript per million (TPM) values. Among the isoforms of contigs, those with the highest TPM values were used, and those with TPM values of 0 were removed. Filtered contigs were subjected to DEG analysis under the condition of FDR < 0.05 using edgeR [44].

### 2.5. Functional Annotation and Gene Set Enrichment Analysis

JEF-197 and JPS beetle contigs were identified using an E-value threshold of 1 × 10^−5^ using the blastx function of Blast2GO v.5.2.5 [45] and the NCBI insect nr database. Immune-related genes in JPS beetle contigs were identified as described above using a database of immune-related genes of arthropods (immunoDB; http://cegg.unige.ch/Insecta/immunodb, accessed on 16 April 2021). Among the contigs identified in the immunoDB, contigs with e-values lower than 1 × 10^−100^ were used to analyze the expression level of immune-related genes. In addition, genes related to Toll, IMD, and JAK/STAT signaling, which are the major immune signaling pathways in insects, were analyzed, including contigs with E-values higher than 1 × 10^−100^. Annotated contigs encoding immune-related genes were classified by immunological class and log_2_ FC value (fungal-treated/non-treated control JPS adults).

Analysis of gene ontology (GO) and KEGG (Kyoto Encyclopedia of Genes and Genomes) pathways was performed by grouping upregulated and downregulated genes. GO analysis of DEGs was performed using the InterProScan function of Blast2GO, which interrogates the CDD, HAMAP, HMMPanther, HMMPfam, HMMPIR, FPrintScan, and BlastProDom databases (https://www.ebi.ac.uk/interpro/, accessed on 16 April 2021). After annotation, we assessed whether upregulated and downregulated contigs belonged to one or more of the following three GO groups: biological process, cellular component, and molecular function at GO level 3. KEGG pathway analysis was performed using the BBH method and insect databases present in the KEGG Automatic Annotation Server (KAAS, http://www.genom.jp/tools/kaas/, accessed on 16 April 2021).

DEGs in fungus-infected JPS beetles were identified based on the *Tribolium castaneum* protein database (Tcas5.2, Ensembl database) and E-value threshold of 1 × 10^−10^ using the blastx function of Blast2GO. DEGs of fungus-infected JPS beetles were classified into eight groups based on up- and downregulated genes (|log_2_FC| < 1, 1–2, 2–3, and >3) and were then subjected to GO enrichment analysis by g:profiler [46] using a Benjamini–Hochberg false discovery rate (FDR) < 0.05 to identify GO terms and KEGG pathways associated with the various groups of genes.

### 2.6. Gene Expression Validation by qRT-PCR

Ten qPCR primers were used in qRT-PCR analyses to validate RNA-seq results. Primers were designed based on the coding regions of contigs using the online program SnapDragon (https://www.flyrnai.org/snapdragon, accessed on 16 April 2021). Sequences of all primers used in this study are listed in Table S1. The actin gene (JPS_TRINITY_DN629_c0_g1) was used as an amplification control. 

Extracted RNA from JPS beetles was used as a template to synthesize cDNA for RT-PCR and qRT-PCR. One microgram of total RNA from each sample was subjected to reverse transcription with an oligo (dT) 15 primer (Promega, MI, USA) using Accupower^®^ RT PreMix (Bioneer, Daejeon, Korea). RT-PCR was conducted using the following conditions: 94 °C for 3 min, 30 cycles of 94 °C for 30 s, 60 °C for 30 s, and 74 °C for 30 s, and a final extension step at 74 °C for 3 min using Accupower ^®^ PCR PreMix (Bioneer, Daejeon, Korea). PCR products were identified by 1.5% agarose gel electrophoresis (data not shown). Real-time PCR was performed using the Thermo Scientific Verso SYBR Green 1-step qRT-PCR ROX Mix kit (Thermo-Fisher Scientific, Carlsbad, CA, USA) and the 96-well Bio-Rad CFX96 Real-Time PCR System (Bio-Rad, Hercules, CA, USA). Total RNA samples that were not reverse transcribed were used as additional negative controls for PCR. PCR conditions were as follows: 95 °C for 2 min followed by 40 cycles of 95 °C for 5 s and 60 °C for 15 s. The actin gene of JPS beetle was used to normalize the expression level of target genes. Melting curve analysis was performed to assess non-specific amplification. Relative gene expression (fold change) was calculated using the 2^−ΔΔCt^ method. All experiments were performed in triplicate. Statistical analyses were performed using Student’s *t*-test, and a *p*-value < 0.05 was considered to indicate a significant difference.

## 3. Results

### 3.1. Time Course of Fungal Virulence

**Figure 1:**
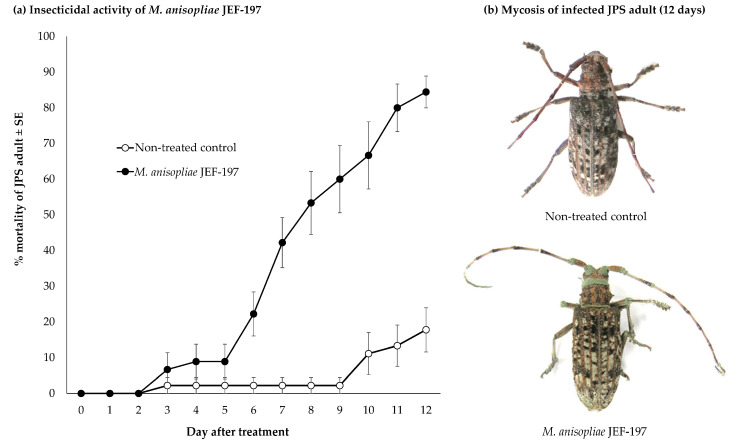
Characteristics of Japanese pine sawyer (JPS) beetle adults treated with the entomopathogenic fungus *M. anisopliae* JEF-197. JPS beetles were inoculated with *M. anisopliae* JEF-197 by spraying of a 1.0 × 10^7^ conidia/mL conidial suspension, and the number of dead JPS adults was counted daily for 12 days. Beetles were maintained at a temperature of 25 ± 2 °C. Non-treated control JPS adults were sprayed with 0.03% siloxane solution. (**a**) Mortality of JPS beetles in response to *M. anisopliae* JEF-197 treatment. (**b**) JPS beetle symptoms induced by fungal infection.

JPS adults sprayed with a conidial suspension (1.0 × 10^7^ conidia/mL) of JEF-197 had a mortality of 53.3% on the 8th day after fungal inoculation (Figure 1a), and the LT_50_ of adults was 7.86 days, which was confirmed by probit analysis (χ^2^ = 8.774, df = 10, *p* = 0.554). On the 10th day after fungal treatment, the mortality of non-treated control and fungus-treated JPS adults was 11.1% and 66.7%, respectively, which was significantly different (*t* = 9.423, *p* < 0.001). On the 4th day after death, white mycelia and green conidia were observed on the surface of JPS cadavers (Figure 1b). After the fungal treatment, the time point of LT_50_ in the infected JPS beetles was determined to extract RNAs and analyze gene expression to figure out the response of beetle when the fungus was actively infecting.

In this work, we analyzed gene expression in JPS beetles and the fungus at the same time points of LT_50_ because the fungus in the infected insect needed to grow enough for analysis. In the results, significant responses of JPS genes against fungal infection were identified; however, the fungal gene expression profile was relatively simple, probably due to the small amounts of fungal transcripts obtained from the infected JPS. Because the immune response is induced at the onset of infection, the immunity of insects at the LT_50_ reflects the late stage of infection. However, it was identified for the persistent immune response of insects against fungal infection. These are described in Section 3.4 and Section 3.6. In order to analyze the reaction to the fungi more clearly, an improvement in the method of the experiment was needed. Previous studies have used transcriptome analysis to investigate interactions based on sequencing of RNA extracted from the pathogen and host at different time points [47]. However, using dual RNA-seq, it is possible to simultaneously measure multiple transcripts without physically separating cells [48]. Individual studies are needed in consideration of the timing of analysis of fungi and insects.

### 3.2. Construction of in Silico cDNA Libraries of M. anisopliae JEF-197 and JAPANESE Pine Sawyer Beetle

A total of 55,816,117 raw RNA-seq reads were obtained from JEF-197 (NCBI SRA accession# PRJNA691966) while 51,085,191 and 50,416,917 raw reads were obtained from negative control and fungus-inoculated JPS beetles (NCBI SRA accession# PRJNA691967), respectively (Table S2). The quality of raw reads was assessed using the fastQC program. The *de novo* assembled transcript sequences of JEF-197 and JPS adults were filtered to select contigs with protein coding capacity; 35,334 and 52,306 contigs including 9286 and 19,046 unigenes were obtained from JEF-197 and JPS beetles, respectively. The N50 of JEF-197 was 1500 bp while that of JPS beetles was 2007 bp (Table S3). JEF-197 and JPS beetles had 59.1% and 65.9% contigs with a contig length less than 1 kb, respectively (Figure S1).

### 3.3. Differentially Expressed Genes in Japanese Pine Sawyer Beetle as a Result of Fungal Infection

**Figure 2:**
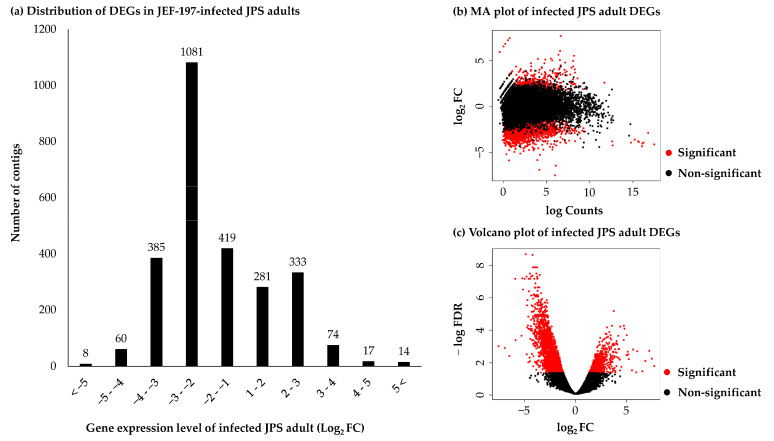
Distribution of differentially expressed genes in JPS adults infected with *M. anisopliae* JEF-197 based on fold change values (FDR < 0.05). The number of contigs for each FC (fold change) value of 1 was counted to assess changes in gene expression of JPS beetles treated with JEF-197. (**a**) Distribution of DEGs in *M. anisopliae* JEF-197-infected JPS adults. (**b**) MA plot of infected JPS adult DEGs. (**c**) Volcano plot of infected JPS adult DEGs.

To accurately measure gene expression levels, only contigs with the highest TPM were retained, and genes with a TPM value of 0 were removed. Finally, 14,156 contigs were filtered for DEG analysis. Illumina short reads of negative control and fungus-treated JPS beetles were mapped to the 14,156 JPS beetle unigenes. Gene expression levels of 2672 JPS beetle unigenes showed significant differences, and there were 719 and 1953 unigenes with increased and decreased expression, respectively. The number of unigenes that were between 2- and 8-fold upregulated or downregulated genes was 614 (85.4%) and 1500 (76.8%), respectively (Figure 2a). Gene expression levels were compared using the condition of FDR < 0.05 in edgeR. There were more unigenes with decreased expression levels than with increased expression levels (Figure 2b,c). To validate the RNA-seq results, the expression levels of seven JPS beetle genes were assessed using qRT-PCR. Gene expression levels measured by qRT-PCR were in a good accordance with those inferred based on RNA-seq (Figure S2).

### 3.4. Changes in Japanese Pine Sawyer Beetle Gene Expression after Fungal Treatment

**Figure 3:**
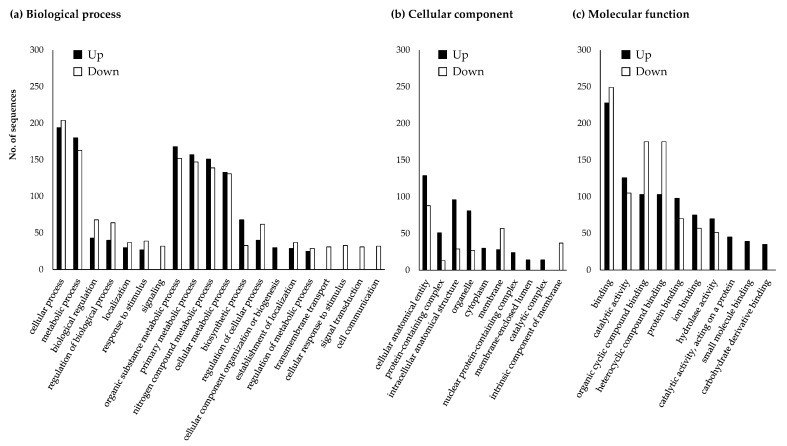
Gene ontology (GO) analysis of DEGs of JPS beetles treated with *M. anisopliae* JEF-197. Unigenes over 2-fold upregulated and less than 0.5-fold downregulated were subjected to analysis using InterProScan. DEGs were identified as being involved in the three GO categories: (**a**) biological process, (**b**) cellular component, and (**c**) molecular function.

Functions of the 2672 DEGs were annotated by InterProScan based on the EMBL database. A total of 578 (48.3%) upregulated and 205 (24.5%) downregulated JPS beetle contigs were annotated with GO functions. As a result, a total 246 and 239 genes were annotated with the GO term of biological process, while 145 and 90 genes were annotated with the GO term cellular component function and 327 and 363 genes with the GO term molecular function, respectively (Figure 3). There were more upregulated unigenes than downregulated unigenes annotated as being involved in biological processes. In particular, many upregulated genes were annotated as being involved in cellular component organization or biogenesis (GO:0071840), while downregulated genes were annotated as having signaling (GO:0023052), transmembrane transport (GO:0055085), cellular response to stimulus (GO:0051716), signal transduction (GO:0007165), and cell communication (GO:0007154) functions (Figure 3a). Most unigenes with a cellular component annotation were upregulated. Only upregulated unigenes were annotated as having a cytoplasmic function (GO:0005737), nuclear protein-containing complex (GO:0140513), membrane-enclosed lumen (GO:0031974), or catalytic complex (GO:1902494) genes, while only downregulated unigenes were annotated as having an intrinsic membrane component (GO:0031224) (Figure 3b). Most unigenes annotated as having a molecular function were upregulated. Only upregulated unigenes were annotated as having catalytic activity (GO:0003824), small molecule binding (GO:0036094), and carbohydrate derivative binding activity (GO:0140096) (Figure 3c).

A total of 414 of 719 upregulated unigenes and 259 of 1953 downregulated JPS beetle unigenes were annotated by a KEGG ontology (KO) identifiers using pathway-based definitions of orthologous genes. KEGG analysis revealed more upregulated pathways than downregulated pathways. The 414 upregulated genes were involved in 250 pathways including the spliceosome (KEGG:03040, 13), RNA transport (KEGG:03013, 13), neurodegeneration-multiple diseases pathways (KEGG:05022, 12), amyotrophic lateral sclerosis (KEGG:05014, 11), Toll and Imd signaling pathways (KEGG:04624, 10), and Shigellosis (KEGG:05131, 10). The 156 downregulated unigenes were involved in 246 pathways including axon regeneration (KEGG:04361, 13), human papillomavirus infection (KEGG:05165, 13), pathways in cancer (KEGG:05200, 12), Wnt signaling pathway (KEGG:04310, 9), axon guidance (KEGG:04360, 9), and the MAPK signaling pathway (KEGG:04010, 8) (Table S4). 

The 652 and 1218 upregulated and downregulated unigenes were annotated using the genome of *T. castaneum*, respectively, and GO enrichment analysis showed that unigenes that were more than 2-fold upregulated were involved in 28 biological processes, 46 cellular components, and 8 molecular functions while unigenes downregulated less than 0.5-fold were involved in 43 biological processes and 10 molecular functions. In the GO enrichment analysis, both up- and downregulated unigenes were annotated with the GO terms of biological processes and molecular function, while only upregulated unigenes received the GO annotation of cellular components. As shown in the GO analysis results, JPS adults infected by JEF-197 showed a decrease in the expression of genes involved in signaling and transmembrane transport. Signaling is critical for information transfer in vivo, while transmembrane transport is important for sequestration of pathogens and manipulation of the transmembrane transport mechanisms of the host [49,50,51]. We predict that signal transmission and transport in JPS beetles are controlled by the fungus upon infection. In particular, pathways affected by the expression of eight gene expression level groups were identified. Genes involved in RNA transport (KEGG:03013) were 2- to 4-fold upregulated. Genes involved in the Toll and Imd signaling pathways (KEGG:04624), ribosome biogenesis in eukaryotes (KEGG:03008), and the spliceosome (KEGG:03040) were 4- to 8-fold upregulated. Genes involved in metabolic pathways (KEGG:01100), cysteine and methionine metabolism (KEGG:00270), glycine, serine, and threonine metabolism (KEGG:00260), purine metabolism (KEGG:00230), one carbon pool by folate (KEGG:00670), and ubiquinone and other terpenoid-quinone biosynthesis (KEGG:00130) pathways were 8-fold upregulated. Genes involved in the hedgehog signaling pathway-fly (KEGG:00670), Wnt signaling pathway (KEGG:00670), and Notch signaling pathway (KEGG:00670) were 0.5- to 0.25-fold downregulated (Table S5).

**Figure 4:**
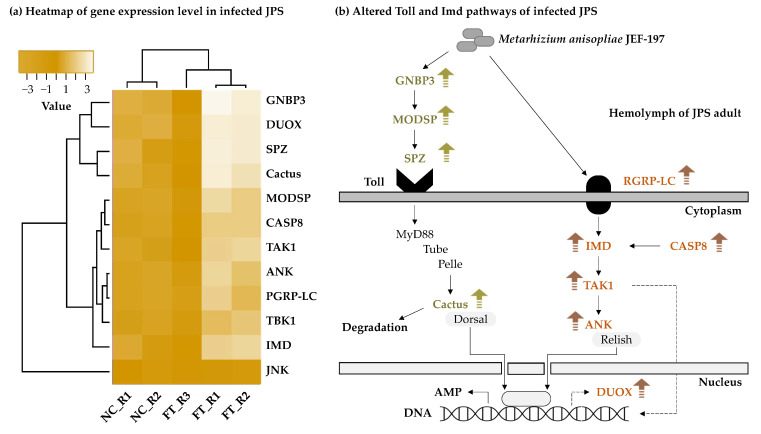
Expression of Toll and IMD pathway-related genes in Japanese pine sawyer adults treated with *M. anisopliae* JEF-197. (**a**) Heatmap of gene expression levels in JPS beetles as a result of fungal treatment. NC, non-treated control JPS adults, and FT, fungus-treated JPS adults. (**b**) Schematic diagram of Toll and Imd pathway genes with altered expression in response to fungal treatment of JPS beetles. GNBP3, Gram-negative bacterial binding protein 3; DUOX, dual oxidase; SPZ, spatzle; Cactus, NF-kappa-B inhibitor alpha; MODSP, modular serine protease; CASP8, caspase 8; TAK1, mitogen-activated protein kinase kinase kinase 7; ANK, ankyrin; PGRP-LC, peptidoglycan recognition protein LC; TBK1, TANK-binding kinase 1; IMD, immune deficiency; JNK, mitogen-activated protein kinase 8/9/10; Toll, protein toll; dMyD88, myeloid differentiation primary response protein MyD88; Tube, interleukin-1 receptor-associated kinase 4; Pelle, interleukin-1 receptor-associated kinase 1; Dorsal, dorsal; Relish, nuclear factor NF-kappa-B p105 subunit.

As mentioned above, certain genes involved in Toll and Imd signaling pathways, which are the main immune signaling pathways in insects, were 4- to 8-fold upregulated. Activation of the Toll and Imd signaling pathways is a typical immune response in fungus-infected insects [52]. Expression levels of the Gram-negative bacteria binding-protein 3 (GNBP3), modular serine protease (ModSP), and Spätzle (Spz) that recognizes β-1,3-glucans of fungi were increased, and the expression levels of Tube, Pelle, and Cactus genes, which encode cytosolic components, were also increased. Expression of IMD pathway gene in JPS adults was increased by JEF-197 treatment. In particular, levels of peptidoglycan recognition protein LC (PGRP-LC), immune deficiency (IMD) protein, and a caspase (CASP8), which are involved in fungal recognition, were upregulated. These two pathways were activated in defensive responses, but genes related to antimicrobial peptides (AMPs) were not annotated (Figure 4a, Table S6). Among DEGs, no genes related to the production of AMPs were annotated. Cecropin and defensin genes, which are AMPs, were identified, but were less than 2-fold upregulated (Table S7). The reason why AMPs were not identified may be because assembly was performed with default settings with the minimum contig length set to 200 bp, and several types of AMPs are short-length sequences. However, expression levels of genes in the Toll and IMD signaling pathway should increase prior to AMP production. Our results indicate that the immune response of JPS beetles was activated in response to fungal infection, and AMP production was likely induced. In previous studies, AMPs such as attacins, cecropin, coleoptericin, and lysozymes were identified in JPS beetles [36]; further studies are needed to determine the repertoire of AMPs produced by JPS beetles in response to fungal infection.

Upregulated genes involved in JPS beetle defense against fungi were assessed by KEGG analysis, and Toll and IMD signaling pathways were identified as playing major roles (Figure 4b, Table S6). The Toll signaling pathway is recognized and initiated by GNBP3, which binds to β-1,3-glucan, a major component of the fungal cell wall [53,54,55]. ModSP activation induces sequential activation of Clip-sectine proteases and Spätzle-processing enzymes [56,57]. Toll is activated by binding of Spätzle cleaved by proteolysis [58]. When Toll is activated, the Toll-interleukin1-resistance (TIR) domain of the Toll receptor recruits a cascade of signaling adapters and kinases to induce phosphorylation of cactus [59]. Toll signaling ultimately induces activation of AMP genes and dissociation of NF-κB protein from cactus [60]. The IMD pathway can be activated as part of the antifungal response, and the Rel/NF-κB transcription factor can be induced in later stages of fungal infection [61,62]. The IMD pathway is activated by recognition of microbial-derived molecules by the peptidoglycan recognition protein LC (PGRP-LC) [63]. Components of the IMD pathway such as Imd, dTAK1, Ird5, Kenny, CASP8, and Relish, which were genes identified as upregulated in our study, regulate the expression of antimicrobial peptide genes by activating Rel/NF-κB-like transcription factors [64]. In addition, the expression of genes involved in the DUOX pathway was increased, suggesting that the bactericidal ROS production pathway was activated in addition to the IMD pathway. Microorganisms capable of destroying DUOX-dependent ROS are regulated by IMD-dependent AMP and may play a complementary role in the ROS system, and ROS plays an important role in the control of intestinal bacteria [65,66].

The Wnt signaling pathway controls the balance between differentiation and proliferation and is involved in the cell cycle and gene transcription [67]. In KEGG pathway analysis, o-palmitoleoyl transferase, which affects both Wnt and Wnt by binding to the frizzled protein to transmit a signal to transcription factor 7 in the cells, was upregulated. In addition, frizzled expression results in gene transcription via MAPK signaling pathway activation. However, genes in this pathway were downregulated, suggesting that Wnt was not recognized by the frizzled receptor. This indicates that the Wnt signaling pathway was not fully functional and that cell cycle and gene transcription activity in JPS beetles was reduced as a result of fungal infection. This is consistent with the decrease in expression of genes with the GO annotation of ‘Signaling’. Downregulated contigs were involved in the Notch signaling pathway, and the expression level of notch was also decreased, suggested decreased activation of the MAPK signaling pathway. Certain genes involved in the MAPK signaling pathway were found to be downregulated, suggesting reduced activation of Wnt and Notch signaling pathways and a decrease in cell cycling and gene transcription [68,69]. Fungal infection appears to reduce the expression of genes in the MAPK signaling pathway, which controls the cell cycle and gene transcription via Wnt and Notch signaling pathways. In our study, active immunity of JPS adults to fungal infection decreased gradually after the eighth day of infection, and infected insects ultimately died. 

### 3.5. Changes in the Expression Level of Japanese Pine Sawyer Beetle Immune-Related Genes

**Figure 5:**
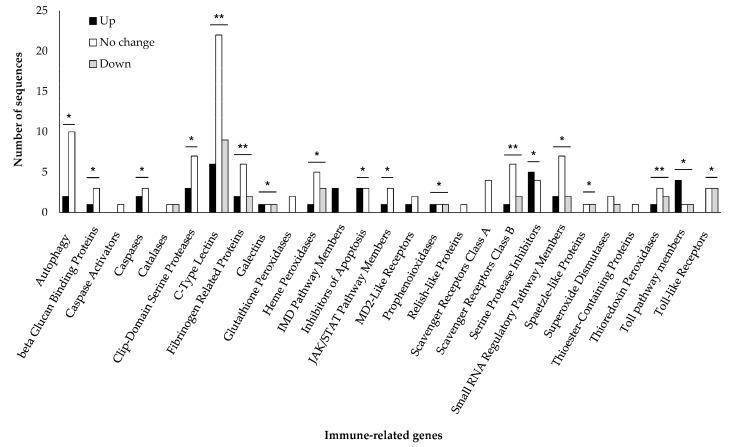
Expression of immune-related genes in JPS beetles in response to *M. anisopliae* JEF-197 fungal treatment. Immune-related genes in JPS beetles whose expression was altered by JEF-197 fungal treatment were determined by Blast2GO based on an arthropod immune-related gene database in immunoDB (E-value < 1.0 × 10^−100^). Genes that showed over 2-fold upregulation or 0.5-fold downregulation were considered upregulated and downregulated, respectively. Differences between groups of gene expression levels in adults with JPS within the immune class were analyzed using a one-way ANOVA in SPSS using a significance level of 0.05 (α). * represents a significant difference in the level of gene expression in the two groups, and ** represents a significant difference in the level of gene expression in the three groups.

Among the differentially expressed JPS beetle unigenes, 172 unigenes were annotated as immune-related genes base on immunoDB (E-value < 1 × 10^−100^) (Table S7). The immune-related genes belonged to 27 immune groups and included genes involved in autophagy, beta glucan binding, as well as genes encoding caspases, clip-domain serine proteases, c-type lectins, IMD pathway members, inhibitors of apoptosis, JAK/STAT pathway members, scavenger receptors class B proteins, serine protease inhibitors, thioredoxin peroxidases, and Toll pathway members (Figure 5). The group of gene expressions in all immune classes, except catalases, MD2-like receptors, and superoxide dissidents, showed significant differences.

Gene expression levels of JPS beetle immune-related unigenes were measured at 6, 24, 48, 72, and 96 h after treatment with JEF-197. Among IMD pathway members, inhibitor of nuclear factor kappa-B kinase gene expression was not significantly altered compared with baseline 6 and 24 h after fungal treatment; however, over 4-fold of upregulation was observed by qRT-PCR at 48 h after treatment, followed by attenuation of the expression level of this gene. Mitogen-activated protein kinase gene expression decreased after fungal infection (Figure S3a,b). Among JAK/STAT pathway members, gene expression of the tyrosine-protein kinase hopscotch decreased slightly up to 24 h after fungal treatment; however, over 1000-fold of upregulation was observed by pRT-PCR at 48 h after treatment, followed by attenuation over time. Cytokine receptor expression continued to decrease after infection, while expression of the signal transducer and activator of transcription (STAT) increased (Figure S3c–e). The JAK/STAT pathway is known to be activated in response to viruses; however, the expression level of STAT increased steadily after treatment. Although this gene is only one of the JAK/STAT genes, a previous study suggested that fungi cannot activate the JAK/STAT pathway. Our result as one of the highlights suggests that fungal infection may induce the expression of JAK/STAT pathway-related genes. Gene expression of Toll-like receptor 7 increased slightly 48 h after fungal treatment and decreased thereafter, while that of the Toll-like receptor Tollo decreased continuously (Figure S3f,g). Among the beta-glucan binding proteins, the expression of beta-1,3-glucan-binding protein (GNBP1) increased after 72 h after treatment as did that of GNBP3 after 24 h after treatment, while the expression of beta-1,3-glucan-binding protein 1 (GNBP2) did not change significantly after 72 h after treatment (Figure S3h,j). Although the change in expression of the Toll-like receptor appears to be insignificant, KEGG enrichment analysis result showed that expression of genes involved in the Toll pathway was increased after fungal treatment; increased expression of GNBP3, which recognizes fungi, may be the trigger that induces activation of the Toll pathway.

### 3.6. Expression of M. anisopliae JEF-197 in Japanese Pine Sawyer Beetles

Three replicate RNA-seq short reads of JEF-197 and fungal-treated JPS beetles were mapped to the contigs of JEF-197. Contigs were filtered, and the filtered contigs were analyzed for DEGs as described above. 

A total of 73 DEGs were identified in JEF-197-infected JPS adults, and 42 of these DEGs were upregulated (Table S8). Of the upregulated contigs, 17 contigs were annotated as functional genes, while 17 hypothetical proteins and 8 no-hit contigs were identified, respectively. Functional genes were involved in energy metabolism, gene transcription regulation, material transport and degradation, and the stress response. 

Energy metabolism-related genes such as oxidoreductase FAD-binding domain protein, ubiquinol-cytochrome c reductase complex subunit, and ATP synthase beta chain precursor were upregulated. Fungi use ATP synthesis to gain energy for invasion of JPS beetles. DNA-directed RNA polymerase Ⅱ, Spt20 family protein, and methyltransferase sirN-like protein, which are genes related to gene transcription regulation, were also upregulated. Recent studies have shown the modulation of pathogenesis and virulence by transcriptional factors of entomopathogenic fungi [70,71,72]. Regulation of activation of gene transcription can contribute to toxicity in the host [73,74], while the recQ family helicase is involved in DNA repair and replication [75]. The increase in expression level of these genes suggests that transcription-, repair-, and replication-related genes play important roles in the pathogenesis and proliferation of the fungus JEF-197 in JPS beetles. The general substrate transporter, which transports small solutes in response to a chemical osmotic ion gradient [76], and the protein BFR2, which regulates protein transport or induces mass cell proliferation [77], were also upregulated in JEF-197.

The response of fungi to stress is essential for fungal growth in an insect host. Superoxide dismutase (SOD) and ubiquitin were identified as upregulated genes. SOD, an enzyme found in all living cells, breaks down superoxide and prevents tissue damage. It also acts as an enzyme that protects against reactive oxygen species (ROS) formed by solar ultraviolet radiation (UA-A and UA-B) and protects conidia against adverse environmental conditions [30,78]. In addition, catalase activity protects against host-derived H_2_O_2_ [79]. In this study, we predicted that the expression of SOD would increase to protect the fungi from H_2_O_2_ produced by JPS beetles. Ubiquitin binds to other proteins and promotes the breakdown of proteins, and its expression is increased in response to stress. In particular, damaged proteins that cannot be recovered due to heat shock are degraded through the ubiquitin–proteasome pathway [80]. We assumed that JEF-197 would have evolved strategies to overcome host-cell-induced stress [81]. In addition, we predicted that this fungus would overcome host-induced stress by increasing energy and protein metabolism and by proliferating in the insect host.

The 42 upregulated DEGs of JEF-197 are candidate genes that likely play crucial roles in invasion of the insect host. Upregulation of genes involved in energy and protein metabolism as well as responses to stressors suggests that these genes are involved in invasion. Defense and proliferation likely occur simultaneously to facilitate the growth of the fungus, and changes in expression patterns of certain fungal genes brought about by methyltransferases likely play a role in toxogenesis in insects. Proteins with unknown functions, such as domain of unknown function (DUF) and hypothetical proteins, showed elevated expression in JPS beetles. The functions of these genes should be clarified in future studies.

In the future, there is a need for practical verification through proteomics and antibody analysis of similar species based on the result of transcriptome analysis. Protein profiles have been proposed as more reliable predictors than transcription profiles [82]. Recently, gene and protein expression studies had mutual connectivity, and transcriptomics data and proteomics data could be complemented by providing protein database and protein level verification, respectively [83,84]. The results of present study, transcriptomic analysis of JPS and *M. anisopliae* that interacted after fungal treatment, will be used as a basis for further study of genomics-inspired proteomics. In the future, we would like to study the correlation between the expression level of the major genes identified in this study and the actual protein production.

## 4. Conclusions

To investigate the interaction between *M. anisopliae* JEF-197 and JPS beetles, we performed RNA-seq analysis of RNA samples obtained from JPS beetles on day 8 after fungal treatment. JPS genes related to RNA transport, ribosome biogenesis, and pathways important for protein production of spliceosomes were upregulated in the JEF-197-infected JPS adults, while JPS genes involved in pathways related to the cell cycle and gene transduction were downregulated. Immune-related genes in the Toll and Imd pathway were upregulated in response to fungal infection, consistent with previous findings. In JEF-197, genes involved in energy, protein metabolism, and stress were upregulated. Although the 42 genes of *M. anisopliae* identified in this study cannot fully explain the pathogenetic mechanisms of this fungus, these 42 genes are candidate genes that play an important role in the fungal invasion to JPS beetles and can provide insight into fungal pathogenesis in future studies.

## Data Availability

The data for this manuscript are available at NCBI SRA, accession number PRJNA691966 and PRJNA691967.

## Supplementary Materials

The following are available online at https://www.mdpi.com/article/10.3390/jof7050373/s1, Figure S1: Distribution of the assembled JPS transcripts according to length of contig, Figure S2: Validation using qRT-PCR for the expression level of the selected seven genes by RNA-seq analysis, Figure S3: The expression levels of immune-related genes in *Metarhizium anisopliae* JEF-197-treated JPS, Table S1: A list of primers used in qRT-PCR to validate the data of RNA-seq analysis and immune-related gene expression in *Metarhizium anisopliae* JEF-197-treated Japanese pine sawyer adult, Table S2: Summary of obtained read of non-treated control and fungus-treated Japanese pine sawyer adults by Illumina sequencing, Table S3: In silico cDNA library of assembled *M. anisopliae* JEF-197 and Japanese pine sawyer, Table S4: KEGG pathway annotation by up- and downregulated unigenes of *Metarhizium anisopliae* JEF-197-treated Japanese pine sawyer, Table S5: Enriched GO term and KEGG of *Metarhizium anisopliae* JEF-197-treated Japanese pine sawyer using a g:profiler, Table S6: DEGs of Japanese pine sawyer treated with *Metarhizium anisopliae* JEF-197 annotated in the IMD and Toll pathway, Table S7: List of immune-related genes in Japanese pine sawyer, Table S8: The DEGs of *Metarhizium anisopliae* JEF-197 in Japanese pine sawyer.

## References

[1] Togashi K. (1989). Development of *Monochamus alternatus* Hope (Coleoptera: Cerambycidae) in relation to oviposition time. Jpn. J. Appl. Entomol. Z..

[2] Burgermeister W., Braasch H., Sousa E., Penas A.C., Mota M., Metge K., Bravo M.A. (1999). First report of *Bursaphelenchus xylophilus* in Portugal and in Europe. Nematology.

[3] Naves P., Mota M., Pires J., Penas A.C., Sousa E., Bonifácio L., Bravo M.A. (2001). *Bursaphelenchus xylophilus* (Nematoda; aphelenchoididae) associated with *Monochamus galloprovincialis* (Coleoptera; Cerambycidae) in Portugal. Nematology.

[4] Lee S., Chung Y., Moon Y., Lee S., Lee D., Choo H., Lee C. (2003). Insecticidal activity and fumigation conditions of several insecticides against Japanese pine sawyer (*Monochamus alternatus*) larvae. J. Korean For. Soc..

[5] Shin S.-C. (2008). Pine wilt disease in Korea. Pine Wilt Disease.

[6] Hemingway J., Ranson H. (2000). Insecticide resistance in insect vectors of human disease. Annu. Rev. Entomol..

[7] Whalon M.E., Mota-Sanchez D., Hollingworth R.M. (2008). Global Pesticide Resistance in Arthropods.

[8] Butt T.M., Jackson C., Magan N., Butt T.M., Jackson C., Magan N. (2001). Introduction-fungal biological control agents: Progress, problems and potential. Fungi as Biocontrol Agents: Progress, Problems and Potential.

[9] Shimazu M. (1994). Potential of the cerambycid-parasitic type of *Beauveria brongniartii* (Deuteromycotina: Hyphomycetes) for microbial control of *Monochamus alternatus* Hope (Coleoptera: Cerambycidae). Appl. Entomol. Zool..

[10] Shimazu M., Kushida T., Tsuchiya D., Mitsuhashi W. (1992). Microbial control of *Monochamus alternatus* Hope (Coleoptera: Cerambycidae) by implanting wheat-bran pellets with *Beauveria bassiana* in infested tree trunks. J. Jpn. For. Soc..

[11] Shimazu M., Sato H. (2003). Effects of larval age on mortality of *Monochamus alternatus* Hope (Coleoptera: Cerambycidae) after application of nonwoven fabric strips with *Beauveria bassiana*. Appl. Entomol. Zool..

[12] Shimazu M., Tsuchiya D., Sato H., Kushida T. (1995). Microbial control of *Monochamus alternatus* Hope (Coleoptera: Cerambycidae) by application of nonwoven fabric strips with *Beauveria bassiana* (Deuteromycotina: Hyphomycetes) on infested tree trunks. Appl. Entomol. Zool..

[13] Francardi V., Rumine P., De Silva J. (2003). On microbial control of *Monochamus galloprovincialis* (Olivier)(Coleoptera Cerambycidae) by means of *Beauveria bassiana* (Bals.) Vuillemin (Deuteromycotina Hyphomycetes). Redia.

[14] Kim J.C., Lee S.J., Kim S., Lee M.R., Baek S., Park S.E., Kim J., Shin T.Y., Kim J.S. (2020). Management of pine wilt disease vectoring *Monochamus alternatus* adults using spray and soil application of *Metarhizium anisopliae* JEF isolates. J. Asia Pac. Entomol..

[15] Kim J.C., Baek S., Park S.E., Kim S., Lee M.R., Jo M., Im J.S., Ha P., Kim J.S., Shin T.Y. (2020). Colonization of *Metarhizium anisopliae* on the surface of pine tree logs: A promising biocontrol strategy for the Japanese pine sawyer, *Monochamus alternatus*. Fungal Biol..

[16] Prior C., Lomer C., Herren H., Paraiso A., Kooyman C., Smit J. The IIBC/IITA/DFPV collaborative research programme on the biological control of locusts and grasshoppers. Proceedings of the Biological Control of Locusts and Grasshoppers: Proceedings of a Workshop Held at the International Institute of Tropical Agriculture.

[17] St Leger R., Joshi L., Bidochka M.J., Roberts D.W. (1996). Construction of an improved mycoinsecticide overexpressing a toxic protease. Proc. Natl. Acad. Sci. USA.

[18] Gillespie and J.P., Kanost M.R., Trenczek T. (1997). Biological mediators of insect immunity. Annu. Rev. Entomol..

[19] Lowenberger C. (2001). Innate immune response of *Aedes aegypti*. Insect Biochem. Molec..

[20] Muta T., Iwanaga S. (1996). The role of hemolymph coagulation in innate immunity. Curr. Opin. Immunol..

[21] Schmidt O., Theopold U., Strand M. (2001). Innate immunity and its evasion and suppression by hymenopteran endoparasitoids. BioEssays.

[22] Strand M.R., Pech L.L. (1995). Immunological basis for compatibility in parasitoid-host relationships. Annu. Rev. Entomol..

[23] Gross J., Müller C., Vilcinskas A., Hilker M. (1998). Antimicrobial Activity of Exocrine Glandular Secretions, Hemolymph, and Larval Regurgitate of the Mustard Leaf Beetle *Phaedon cochleariae*. J. Invertebr. Pathol..

[24] Boucias D., Latgé J. (1988). Nonspecific induction of germination of *Conidiobolus obscurus* and *Nomuraea rileyi* with host and non-host cuticle extracts. J. Invertebr. Pathol..

[25] Holder D.J., Keyhani N.O. (2005). Adhesion of the entomopathogenic fungus *Beauveria* (*Cordyceps*) *bassiana* to substrata. Appl. Environ. Microb..

[26] Wraight S., Butt T., Galaini-Wraight S., Allee L., Soper R., Roberts D.W. (1990). Germination and infection processes of the entomophthoralean fungus *Erynia radicans* on the potato leafhopper, *Empoasca fabae*. J. Invertebr. Pathol..

[27] Zipfel P.F., Skerka C., Kupka D., Luo S. (2011). Immune escape of the human facultative pathogenic yeast *Candida albicans*: The many faces of the *Candida* Pra1 protein. Int. J. Med. Microbiol. Suppl..

[28] Brey P.T., Lee W.-J., Yamakawa M., Koizumi Y., Perrot S., Francois M., Ashida M. (1993). Role of the integument in insect immunity: Epicuticular abrasion and induction of cecropin synthesis in cuticular epithelial cells. Proc. Natl. Acad. Sci. USA.

[29] Butt T.M., Hoch H.C., Staples R.C., Leger R.J.S. (1989). Use of fluorochromes in the study of fungal cytology and differentiation. Exp. Mycol..

[30] Santi L., da Silva W.O.B., Berger M., Guimarães J.A., Schrank A., Vainstein M.H. (2010). Conidial surface proteins of *Metarhizium anisopliae*: Source of activities related with toxic effects, host penetration and pathogenesis. Toxicon.

[31] Schrank A., Vainstein M.H. (2010). *Metarhizium anisopliae* enzymes and toxins. Toxicon.

[32] Birzele F., Schaub J., Rust W., Clemens C., Baum P., Kaufmann H., Weith A., Schulz T.W., Hildebrandt T. (2010). Into the unknown: Expression profiling without genome sequence information in CHO by next generation sequencing. Nucleic Acids Res..

[33] Feng H., Qin Z., Zhang X. (2013). Opportunities and methods for studying alternative splicing in cancer with RNA-Seq. Cancer Lett..

[34] McGettigan P.A. (2013). Transcriptomics in the RNA-seq era. Curr. Opin. Chem. Biol..

[35] Lin T., Cai Z., Wu H. (2015). Transcriptome analysis of the Japanese pine sawyer beetle, *Monochamus alternatus* (Coleoptera: Cerambycidae) by high-throughput Illumina sequencing. J. Asia Pac. Entomol..

[36] Zhou J., Yu H.Y., Zhang W., Ahmad F., Hu S.N., Zhao L.L., Zou Z., Sun J.H. (2018). Comparative analysis of the *Monochamus alternatus* immune system. Insect Sci..

[37] Wu S., Zhu X., Liu Z., Shao E., Rebeca C.-L., Guo Y., Xiong Y., Mou Y., Xu R., Hu X. (2016). Identification of genes relevant to pesticides and biology from global transcriptome data of *Monochamus alternatus* Hope (Coleoptera: Cerambycidae) larvae. PLoS ONE.

[38] Wang J., Li D.-Z., Min S.-F., Mi F., Zhou S.-S., Wang M.-Q. (2014). Analysis of chemosensory gene families in the beetle *Monochamus alternatus* and its parasitoid *Dastarcus helophoroides*. Comp. Biochem. Physiol. Part. D Genom. Proteom..

[39] Zhang W., Meng J., Ning J., Qin P., Zhou J., Zou Z., Wang Y., Jiang H., Ahmad F., Zhao L. (2017). Differential immune responses of *Monochamus alternatus* against symbiotic and entomopathogenic fungi. Sci. China Life Sci..

[40] Andrews S. (2010). FastQC: A Quality Control Tool for High Throughput Sequence Data. http://www.bioinformatics.babraham.ac.uk/projects/fastqc/.

[41] Bolger A.M., Lohse M., Usadel B. (2014). Trimmomatic: A flexible trimmer for Illumina sequence data. Bioinformatics.

[42] Haas B.J., Papanicolaou A., Yassour M., Grabherr M., Blood P.D., Bowden J., Couger M.B., Eccles D., Li B., Lieber M. (2013). *De novo* transcript sequence reconstruction from RNA-seq using the Trinity platform for reference generation and analysis. Nat. Protoc..

[43] Bray N.L., Pimentel H., Melsted P., Pachter L. (2016). Near-optimal probabilistic RNA-seq quantification. Nat. Biotechnol..

[44] Robinson M.D., McCarthy D.J., Smyth G.K. (2010). edgeR: A Bioconductor package for differential expression analysis of digital gene expression data. Bioinformatics.

[45] Conesa A., Götz S., García-Gómez J.M., Terol J., Talón M., Robles M. (2005). Blast2GO: A universal tool for annotation, visualization and analysis in functional genomics research. Bioinformatics.

[46] Reimand J., Arak T., Adler P., Kolberg L., Reisberg S., Peterson H., Vilo J. (2016). g: Profiler—A web server for functional interpretation of gene lists (2016 update). Nucleic Acids Res..

[47] Oosthuizen J.L., Gomez P., Ruan J., Hackett T.L., Moore M.M., Knight D.A., Tebbutt S.J. (2011). Dual organism transcriptomics of airway epithelial cells interacting with conidia of *Aspergillus fumigatus*. PLoS ONE.

[48] Westermann A.J., Gorski S.A., Vogel J. (2012). Dual RNA-seq of pathogen and host. Nat. Rev. Microbiol..

[49] Heinz E., Hacker C., Dean P., Mifsud J., Goldberg A.V., Williams T.A., Nakjang S., Gregory A., Hirt R.P., Lucocq J.M. (2014). Plasma membrane-located purine nucleotide transport proteins are key components for host exploitation by microsporidian intracellular parasites. PLoS Pathog..

[50] Cossart P., Roy C.R. (2010). Manipulation of host membrane machinery by bacterial pathogens. Curr. Opin. Cell Biol..

[51] Alix E., Mukherjee S., Roy C.R. (2011). Subversion of membrane transport pathways by vacuolar pathogens. J. Cell Biol..

[52] Tanji T., Ip Y.T. (2005). Regulators of the Toll and Imd pathways in the *Drosophila* innate immune response. Trends Immunol..

[53] Lee S.H., Carpenter J.F., Chang B.S., Randolph T.W., Kim Y.S. (2006). Effects of solutes on solubilization and refolding of proteins from inclusion bodies with high hydrostatic pressure. Protein Sci..

[54] Ma C., Kanost M.R. (2000). A β1, 3-glucan recognition protein from an insect, *Manduca sexta*, agglutinates microorganisms and activates the phenoloxidase cascade. J. Biol. Chem..

[55] Ochiai M., Ashida M. (2000). A pattern-recognition protein for β-1, 3-glucan: The binding domain and the cDNA cloning of β-1, 3-glucan recognition protein from the silkworm, *Bombyx mori*. J. Biol. Chem..

[56] Buchon N., Poidevin M., Kwon H.-M., Guillou A., Sottas V., Lee B.-L., Lemaitre B. (2009). A single modular serine protease integrates signals from pattern-recognition receptors upstream of the *Drosophila* Toll pathway. Proc. Natl. Acad. Sci. USA.

[57] Gobert V., Gottar M., Matskevich A.A., Rutschmann S., Royet J., Belvin M., Hoffmann J.A., Ferrandon D. (2003). Dual activation of the *Drosophila* toll pathway by two pattern recognition receptors. Science.

[58] Ligoxygakis P., Pelte N., Hoffmann J.A., Reichhart J.-M. (2002). Activation of *Drosophila* Toll during fungal infection by a blood serine protease. Science.

[59] Hoffmann J.A., Reichhart J.-M. (2002). *Drosophila* innate immunity: An evolutionary perspective. Nat. Immunol..

[60] Hoffmann J.A. (2003). The immune response of *Drosophila*. Nature.

[61] Hedengren-Olcott M., Olcott M.C., Mooney D.T., Ekengren S., Geller B.L., Taylor B.J. (2004). Differential activation of the NF-κB-like factors Relish and Dif in *Drosophila melanogaster* by fungi and Gram-positive bacteria. J. Biol. Chem..

[62] Ramirez J.L., Dunlap C.A., Muturi E.J., Barletta A.B., Rooney A.P. (2018). Entomopathogenic fungal infection leads to temporospatial modulation of the mosquito immune system. PLoS Negl. Trop. Dis..

[63] Hedengren M., Dushay M.S., Ando I., Ekengren S., Wihlborg M., Hultmark D. (1999). Relish, a central factor in the control of humoral but not cellular immunity in *Drosophila*. Mol. Cell.

[64] Stöven S., Silverman N., Junell A., Hedengren-Olcott M., Erturk D., Engström Y., Maniatis T., Hultmark D. (2003). Caspase-mediated processing of the *Drosophila* NF-κB factor Relish. Proc. Natl. Acad. Sci. USA.

[65] Ryu J.-H., Ha E.-M., Lee W.-J. (2010). Innate immunity and gut–microbe mutualism in *Drosophila*. Dev. Comp. Immunol..

[66] Bae Y.S., Choi M.K., Lee W.-J. (2010). Dual oxidase in mucosal immunity and host–microbe homeostasis. Trends Immunol..

[67] Clevers H., Loh K.M., Nusse R. (2014). An integral program for tissue renewal and regeneration: Wnt signaling and stem cell control. Science.

[68] Wodarz A., Nusse R. (1998). Mechanisms of Wnt signaling in development. Annu. Rev. Cell Dev. Biol..

[69] Artavanis-Tsakonas S., Rand M.D., Lake R.J. (1999). Notch signaling: Cell fate control and signal integration in development. Science.

[70] Xin C., Yang J., Mao Y., Chen W., Wang Z., Song Z. (2020). GATA-type transcription factor MrNsdD regulates dimorphic transition, conidiation, virulence and microsclerotium formation in the entomopathogenic fungus *Metarhizium rileyi*. Microb. Biotechnol..

[71] Wang J.J., Yin Y.P., Song J.Z., Hu S.J., Cheng W., Qiu L. (2021). A p53-like transcription factor, BbTFO1, contributes to virulence and oxidative and thermal stress tolerances in the insect pathogenic fungus, *Beauveria bassiana*. PLoS ONE.

[72] Zhao X., Luo T., Huang S., Peng N., Yin Y., Luo Z., Zhang Y. (2021). A novel transcription factor negatively regulates antioxidant response, cell wall integrity and virulence in the fungal insect pathogen, *Beauveria bassiana*. Environ. Microbiol..

[73] Wang Y., Wang T., Qiao L., Zhu J., Fan J., Zhang T., Wang Z.-x., Li W., Chen A., Huang B. (2017). DNA methyltransferases contribute to the fungal development, stress tolerance and virulence of the entomopathogenic fungus *Metarhizium robertsii*. Appl. Microbiol. Biotechnol..

[74] Lei B., Zhou N., Guo Y., Zhao W., Tan Y.-W., Yu Y., Lu H. (2014). Septin ring assembly is regulated by Spt20, a structural subunit of the SAGA complex. J. Cell Sci..

[75] Anderson R.M., Sinclair D.A., Lebel M. (2004). Yeast RecQ Helicases: Clues to DNA Repair, Genome Stability and Aging. Molecular Mechanisms of Werner’s Syndrome.

[76] Saier M.H. (1998). Molecular phylogeny as a basis for the classification of transport proteins from bacteria, archaea and eukarya. Adv. Mircrob. Physiol..

[77] Chabane S., Gachet E., Képès F. (1998). Over-expression of the yeast BFR2 gene partially suppresses the growth defects induced by Brefeldin A and by four ER-to-Golgi mutations. Curr. Genet..

[78] Rangel D.E., Braga G.U., Flint S.D., Anderson A.J., Roberts D.W. (2004). Variations in UV-B tolerance and germination speed of *Metarhizium anisopliae* conidia produced on insects and artificial substrates. J. Invertebr. Pathol..

[79] Garre V., Tenberge K.B., Eising R. (1998). Secretion of a fungal extracellular catalase by *Claviceps purpurea* during infection of rye: Putative role in pathogenicity and suppression of host defense. Phytopathology.

[80] Shang F., Taylor A. (2011). Ubiquitin–proteasome pathway and cellular responses to oxidative stress. Free Radic. Biol. Med..

[81] Wang Z.-X., Zhou X.-Z., Meng H.-M., Liu Y.-J., Zhou Q., Huang B. (2014). Comparative transcriptomic analysis of the heat stress response in the filamentous fungus *Metarhizium anisopliae* using RNA-Seq. Appl. Microbiol. Biotechnol..

[82] Diz A.P., Martínez-fernández M., Rolán-alvarez E. (2012). Proteomics in evolutionary ecology: Linking the genotype with the phenotype. Mol. Ecol..

[83] Nesvizhskii A.I. (2014). Proteogenomics: Concepts, applications and computational strategies. Nat. Methods.

[84] Lopez-Casado G., Covey P.A., Bedinger P.A., Mueller L.A., Thannhauser T.W., Zhang S., Fei Z., Giovannoni J.J., Rose J.K. (2012). Enabling proteomic studies with RNA-Seq: The proteome of tomato pollen as a test case. Proteomics.

