# Supplementary material for: Transcriptome Analysis of the Japanese Pine Sawyer Beetle, Monochamus alternatus, Infected with the Entomopathogenic Fungus Metarhizium anisopliae JEF-197
Source: J Fungi (Basel). 2021 May 10;7(5):373. doi: 10.3390/jof7050373 (PMC8151162; doi:10.3390/jof7050373)
Supplement: Supplementary file 1 [file jof-07-00373-s001.zip › Supplementary Figure S1.pdf]

(a) *Metarhizium anisopliae* JEF-197

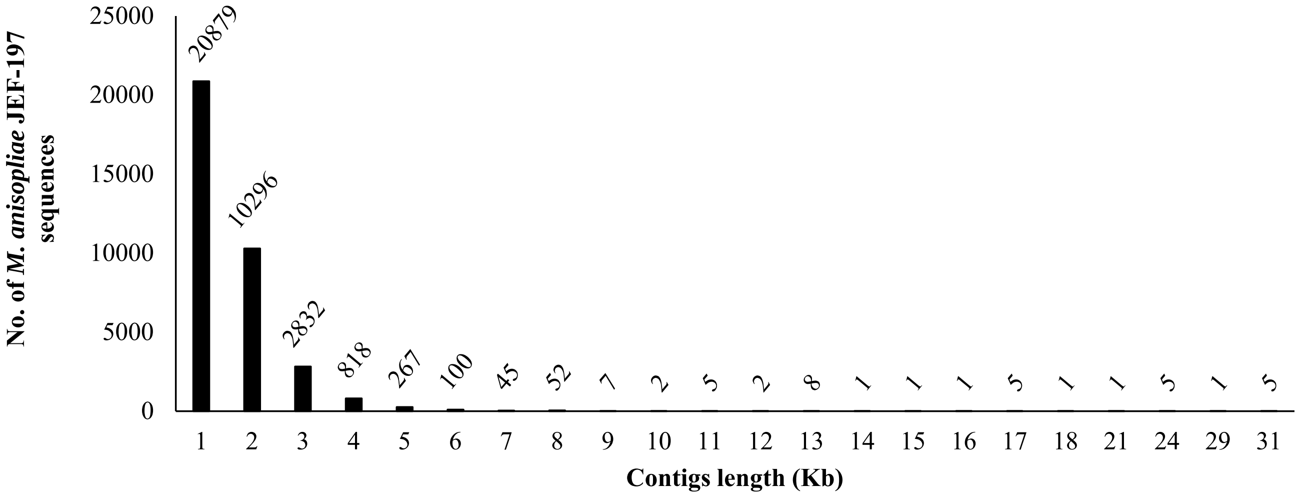

(b) Japanese pine sawyer beetle

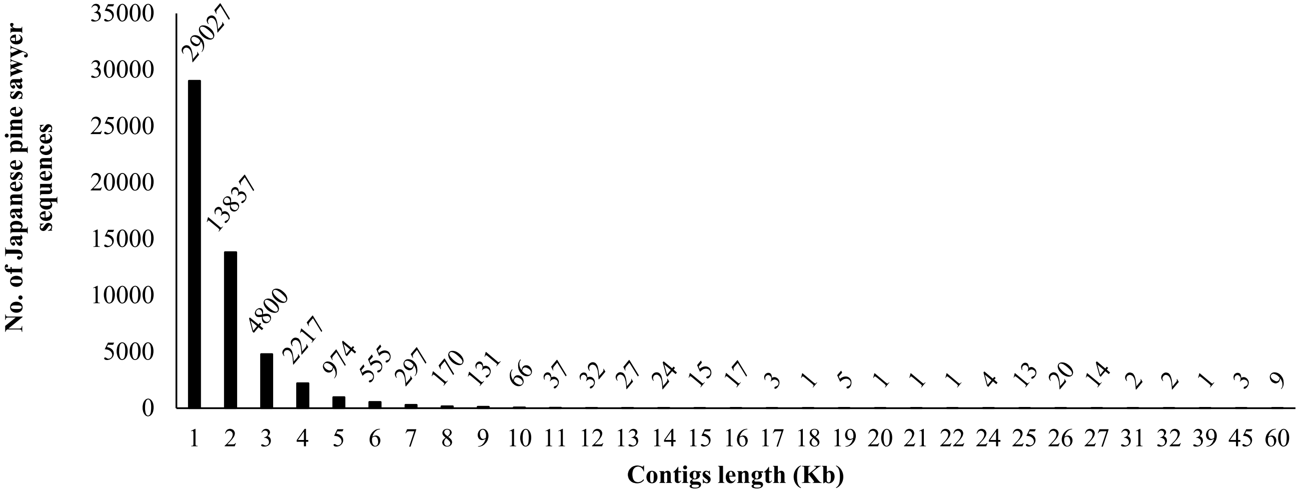

Supplementary Figure S1. Distribution of the assembled Japanese pine sawyer beetle transcripts according to length of contig The number of contigs was counted between the corresponding 1 kb size criteria. (a), The contigs of *Metarhizium anisopliae* JEF-197; (b), The contigs of JPS beetle
