# Supplementary material for: Transcriptome Analysis of the Japanese Pine Sawyer Beetle, Monochamus alternatus, Infected with the Entomopathogenic Fungus Metarhizium anisopliae JEF-197
Source: J Fungi (Basel). 2021 May 10;7(5):373. doi: 10.3390/jof7050373 (PMC8151162; doi:10.3390/jof7050373)
Supplement: Supplementary file 1 [file jof-07-00373-s001.zip › Supplementary Figure S2.pdf]

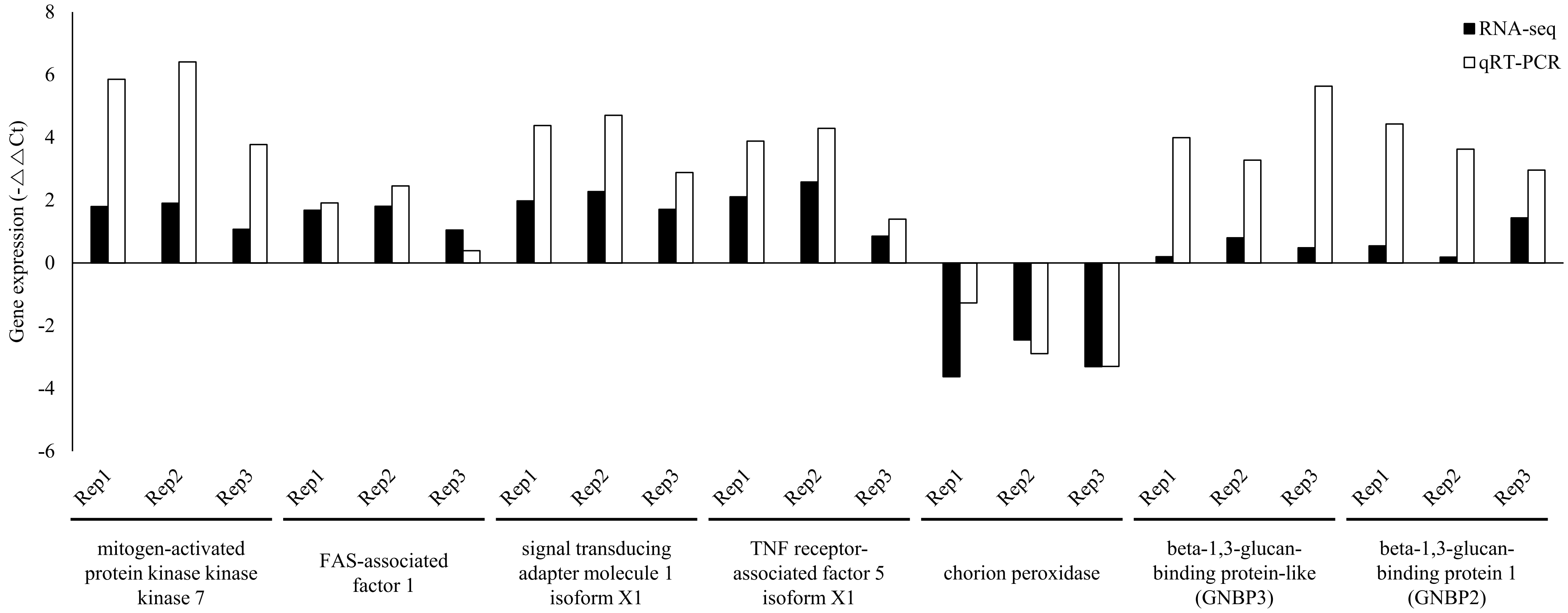

Supplementary Figure S2. Validation using qRT-PCR for the expression level of the selected seven genes by RNA-seq analysis. The *actin* gene (JPS_TRINITY_DN629_c0_g1) was used as internal control of Japanese pine sawyer. The average results of three replicates RNA samples of the non-treated control Japanese pine sawyer were compared with each results of the fungus-treated JPS. The Rep is replicates of fungus-treated JPS beetle.
