# Supplementary material for: Transcriptome Analysis of the Japanese Pine Sawyer Beetle, Monochamus alternatus, Infected with the Entomopathogenic Fungus Metarhizium anisopliae JEF-197
Source: J Fungi (Basel). 2021 May 10;7(5):373. doi: 10.3390/jof7050373 (PMC8151162; doi:10.3390/jof7050373)
Supplement: Supplementary file 1 [file jof-07-00373-s001.zip › Supplementary Figure S3.pdf]

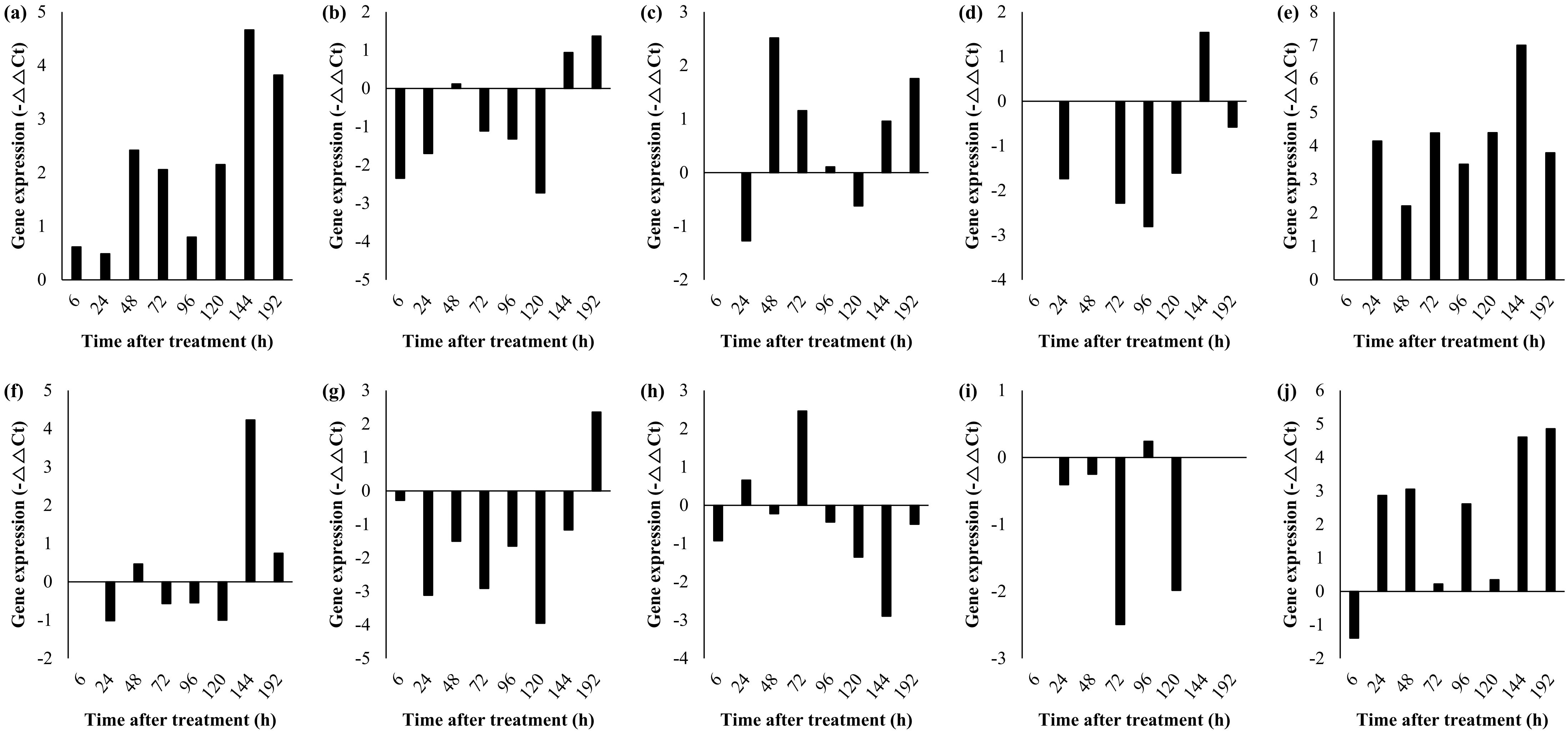

Supplementary Figure S3. The expression levels of immune-related genes in *Metarhizium anisopliae* JEF-197-treated JPS. The fungi were treated on JPS by spray conidia suspension at 1.0×10^7 conidia/ml of *M. anisopliae* JEF-197. (a), inhibitor of nuclear factor kappa-B kinase; (b), mitogen-activated protein kinase; (c), tyrosine-protein kinase hopscotch; (d), cytokine receptor; (e), signal transducer and activator of transcription; (f), toll-like receptor 7; (g), toll-like receptor Tollo; (h), beta-1,3-glucan-binding protein (GNBP1); (i) beta-1,3-glucan-binding protein 1 (GNBP2); and (j), beta-1,3-glucan-binding protein-like (GNBP3). The *actin* gene (JPS_TRINITY_DN629_c0_g1) was used as internal control of Japanese pine sawyer.
