# Supplementary material for: Transcriptome Analysis of the Japanese Pine Sawyer Beetle, Monochamus alternatus, Infected with the Entomopathogenic Fungus Metarhizium anisopliae JEF-197
Source: J Fungi (Basel). 2021 May 10;7(5):373. doi: 10.3390/jof7050373 (PMC8151162; doi:10.3390/jof7050373)
Supplement: Supplementary file 1 [file jof-07-00373-s001.zip › Supplementary Table S1.pdf]

Supplementary Table S1. A list of primers used in qRT-PCR to validate the data of RNA-seq analysis and immune-related gene expression in *Metarhizium anisopliae* JEF-197-treated Japanese pine sawyer beetle adult

Transcripts	Gene	Amplicon size (bp)		Primer (5’ → 3′)
TRINITY_DN629_c0_g1_i5	Actin-87E-like Protein, actin, muscle (internal control)	196	F	TGC GAC GTT GAT ATC CGT AA
			R	AGA GGG AAG CCA AGA TGG AT
TRINITY_DN3896_c0_g1_i4	inhibitor of nuclear factor kappa-B kinase subunit alpha-like isoform X1 (IMD pathway members)	224	F	CGA AAA ATG CTT CCG GAT TA
			R	ACC CAC AAA GCT CAC TAC CG
TRINITY_DN3275_c0_g1_i2	mitogen-activated protein kinase kinase kinase 7 (IMD pathway members)	174	F	GCT AAT GCT TGG GAT TTG GA
			R	ATC CAG GGA CTC TAG CAG CA
TRINITY_DN5026_c0_g1_i2	tyrosine-protein kinase hopscotch (JAK/STAT pathway members)	165	F	CAG CAG CTT TGT GGC ACT TA
			R	CCA CTG GGA TCC AAT GTA CC
TRINITY_DN1489_c0_g1_i3	cytokine receptor (JAK/STAT pathway members)	241	F	TGA AGT CAG TTG GCA AGC AC
			R	ACA TTC AGC CCA AAA CAT CC
TRINITY_DN191_c0_g2_i6	signal transducer and activator of transcription 5B isoform X1 (JAK/STAT pathway members)	193	F	AAT ACC AAC AGG CGA CAA GG
			R	ACC ACG ACG GGT AAA CTC AG
TRINITY_DN5529_c0_g1_i1	toll-like receptor 7 (Toll-like receptors)	241	F	CGT AAT CTC CGT GTC CGA TT
			R	CAC CAC CCA TTC TTC GTC TT
TRINITY_DN61089_c0_g1_i1	toll-like receptor Tollo (Toll-like receptors)	209	F	TGC CGT GCT AGA TTC TTC CT
			R	GTA TGA CTT GGC GCT GGA TT
TRINITY_DN7214_c0_g1_i1	beta-1,3-glucan-binding protein (GNBP1)	157	F	AGA AGG TGG CTT TTG GGA AT
			R	AGC ATC ATC GGG GAA GTA TG
TRINITY_DN8099_c1_g1_i2	beta-1,3-glucan-binding protein-like (GNBP3)	211	F	TGA TAT TTA GCG AGA GTT TT
			R	TTG GCG ACA TTA CAA ATC CA
TRINITY_DN7462_c0_g1_i1	beta-1,3-glucan-binding protein 1 (GNBP2)	197	F	GCC TGG AGA TTT CAG TGG AG
			R	CCT CAC AAC CTG TTC CTG GT
