# Supplementary material for: Transcriptome Analysis of the Japanese Pine Sawyer Beetle, Monochamus alternatus, Infected with the Entomopathogenic Fungus Metarhizium anisopliae JEF-197
Source: J Fungi (Basel). 2021 May 10;7(5):373. doi: 10.3390/jof7050373 (PMC8151162; doi:10.3390/jof7050373)
Supplement: Supplementary file 1 [file jof-07-00373-s001.zip › Supplementary Table S2.pdf]

Supplementary Table S2. Summary of obtained read of non-treated control and fungus-treated Japanese pine sawyer adults by Illumina sequencing

**Treatment**	***M. anisopliae* JEF-197**			**Non-treated control JPS**			**Fungus-treated JPS**		
**Replication**	1	2	3	1	2	3	1	2	3
**Total number of sequences**	15,158,641	16,588,362	24,069,114	18,329,493	17,487,218	15,268,480	15,390,184	15,487,044	19,539,689
**Total length (bp)**	3,062,045,482	3,350,849,124	4,861,961,028	3,702,557,586	3,532,418,036	3,084,232,960	3,108,817,168	3,128,382,888	3,947,017,178
**GC%**	53	52	52	51	43	49	44	43	44
