# Supplementary material for: Transcriptome Analysis of the Japanese Pine Sawyer Beetle, Monochamus alternatus, Infected with the Entomopathogenic Fungus Metarhizium anisopliae JEF-197
Source: J Fungi (Basel). 2021 May 10;7(5):373. doi: 10.3390/jof7050373 (PMC8151162; doi:10.3390/jof7050373)
Supplement: Supplementary file 1 [file jof-07-00373-s001.zip › Supplementary Table S3.pdf]

Supplementary Table S3. *in silico* cDNA library of assembled *M. anisopliae* JEF-197 and Japanese pine sawyer

	M. anisopliae JEF-197	Japanese pine sawyer
Total length of transcripts (bp)	38,664,210	71,371,911
Total number of transcripts	35,334	52,306
Total number of genes	9,286	19,046
N50 of genes (bp)	1,500	2,007
GC %	54.19	41.74
