# Supplementary material for: Transcriptome Analysis of the Japanese Pine Sawyer Beetle, Monochamus alternatus, Infected with the Entomopathogenic Fungus Metarhizium anisopliae JEF-197
Source: J Fungi (Basel). 2021 May 10;7(5):373. doi: 10.3390/jof7050373 (PMC8151162; doi:10.3390/jof7050373)
Supplement: Supplementary file 1 [file jof-07-00373-s001.zip › Supplementary Table S6.pdf]

Supplementary Table S6. DEGs of Japanese pine sawyer treated with *Metarhizium anisopliae* JEF-197 annotated in the IMD and Toll pathway

Pathway	Name	Definition	KO entry	Contigs
IMD pathway	PGRP-LC	peptidoglycan recognition protein LC	K24075	JPS_TRINITY_DN9_c0_g2
	IMD	immune deficiency	K20699	JPS_TRINITY_DN143931_c0_g1
	ANK	ankyrin	K10380	JPS_TRINITY_DN588_c0_g1
	DUOX	dual oxidase [EC:1.6.3.1 1.11.1.-]	K13411	JPS_TRINITY_DN1347_c0_g1
	MAP3K7	mitogen-activated protein kinase kinase kinase 7 [EC:2.7.11.25]	K04427	JPS_TRINITY_DN3275_c0_g1
	PGRP	peptidoglycan recognition protein	K01446	JPS_TRINITY_DN6549_c1_g2
	CASP8 (Dredd)	caspase 8 [EC:3.4.22.61]	K04398	JPS_TRINITY_DN5698_c0_g1
Toll pathway	GNBP3	gram-negative bacteria-binding protein 3	K20692	JPS_TRINITY_DN2475_c0_g2
	MODSP	modular serine protease [EC:3.4.21.-]	K20674	JPS_TRINITY_DN2050_c0_g1
	SPZ	protein spaetzle	K20694	JPS_TRINITY_DN5430_c0_g1
	NFKBIA	NF-kappa-B inhibitor alpha	K04734	JPS_TRINITY_DN3708_c0_g1
Toll-like receptor signaling pathway	CASP8	caspase 8 [EC:3.4.22.61]	K04398	JPS_TRINITY_DN5698_c0_g1
	TBK1	TANK-binding kinase 1 [EC:2.7.11.10]	K05410	JPS_TRINITY_DN6262_c0_g1
	MAP3K7	mitogen-activated protein kinase kinase kinase 7 [EC:2.7.11.25]	K04427	JPS_TRINITY_DN3275_c0_g1
	NFKBIA	caspase 8 [EC:3.4.22.61]	K04398	JPS_TRINITY_DN3708_c0_g1
